# Dopamine, norepinephrine, and vasopressin accelerate particle transport velocity in murine tracheal epithelium via substance-specific receptor pathways: dependency on intra- and extracellular Ca^2+^ sources

**DOI:** 10.3389/fphar.2024.1401983

**Published:** 2024-09-06

**Authors:** Götz Schmidt, Isabelle Greif, Sabrina Müller, Melanie Markmann, Fabian Edinger, Michael Sander, Christian Koch, Michael Henrich

**Affiliations:** ^1^ Department of Anesthesiology, Intensive Care Medicine and Pain Therapy, Justus Liebig University Giessen, Giessen, Germany; ^2^ Department of Anesthesiology, Intensive Care Medicine, Emergency Medicine, Vidia St. Vincentius-Clinic Karlsruhe gAG, Karlsruhe, Germany

**Keywords:** vasopressor, mucociliary clearance, ciliary beat frequency, perioperative, ciliary activity

## Abstract

**Background:**

The unique ability of the respiratory tract to protect the integrity of the airways by removing potentially harmful substances is defined as mucociliary clearance. This complex physiological mechanism protects the lower airways by ridding them of pollutants and pathogens. This study aimed to evaluate the potential influence of clinically relevant vasopressors on mucociliary clearance.

**Material and methods:**

The particle transport velocity (PTV) of isolated murine tracheae was measured as a surrogate for mucociliary clearance under the influence of dopamine, norepinephrine, and vasopressin. Inhibitory substances were applied to elucidate relevant signal transduction cascades and the value and origin of calcium ions. Reverse-transcription polymerase chain reactions (RT-PCR) were performed to identify the expression of vasopressin receptor subtypes.

**Results:**

Dopamine, norepinephrine, and vasopressin significantly increased the PTV in a dose-dependent manner with half maximal effective concentrations of 0.58 µM, 1.21 µM, and 0.10 µM, respectively. Each substance increased the PTV via separate receptor pathways. While dopamine acted on D_1_-like receptors to increase the PTV, norepinephrine acted on β-adrenergic receptors, and vasopressin acted on V_1a_ receptors. RT-PCR revealed the expression of V_1a_ in the murine whole trachea and tracheal epithelium. PTV increased when protein kinase A was inhibited and norepinephrine or vasopressin were applied, but not when dopamine was applied. Phospholipase C inhibition decreased the PTV when vasopressin was applied. In general, maximum PTV was significantly reduced when extracellular calcium entry was inhibited. When intracellular calcium stores were depleted, no increase in PTV was observed after administering all three substances. Inositol trisphosphate receptor activation was found to be pivotal in the increase in murine PTV after applying dopamine and vasopressin.

**Discussion:**

Dopamine, norepinephrine, and vasopressin accelerate the murine PTV via substance-specific receptor pathways. Further investigations should assess the value and interaction of these substances on mucociliary clearance in clinical practice.

## 1 Introduction

The unique ability of the respiratory tract to protect the integrity of the airways by removing potentially harmful substances is called mucociliary clearance. Mucociliary clearance protects the lungs by ridding the airways of pollution and preventing pathogen colonization, and infection. The cooperation of different cells, such as basal, suprabasal, goblet, and multiciliated epithelial cells, ensures the complex mucociliary clearance function of the respiratory tract (Lee and Foskett, 2014; Legendre et al., 2021). These ciliated epithelial cells use outward-directed transportation to prevent the accumulation of debris and colonization by microbial pathogens (Whitsett, 2018; Legendre et al., 2021). Cilia are located on the apical side of the respiratory epithelium along the airways, and are constructed from unique structural proteins (Whitsett, 2018). Ciliary activity can be quantified as ciliary beat frequency (CBF), with particle transport velocity (PTV) used as its surrogate (Delmotte and Sanderson, 2006; Weiterer et al., 2014).

Mucociliary clearance is a complex process that can adapt to different physiological and pathophysiological conditions, with many endogenous and exogenous pathways involved in modifying its function (Delmotte and Sanderson, 2006; Legendre et al., 2021). Therefore, not only inhaled particles but also mucus, electrolytes, and endogenous defensive substances can be removed from the lower airways, which are then expectorated under normal physiological conditions (Delmotte and Sanderson, 2006; Legendre et al., 2021).

The sympathetic and parasympathetic nervous systems increase or decrease ciliary activity, facilitated by their transmitters, norepinephrine and acetylcholine (Frohock et al., 2002; Hollenhorst and Krasteva-Christ, 2021). Important secondary messengers are cyclic adenosine monophosphate (cAMP), cyclic guanosine monophosphate, and calcium ions (Ca^2+^), which ultimately induce alterations in the CBF (Salathe, 2007; Wyatt, 2015). However, ciliary activity is also sensitive to local temperature, acid-base balance, humidity, mechanical stress, cytokines released during infection, and paracrine effects mediated by adjacent cells, such as mast cells or macrophages (Sanderson and Dirksen, 1986; Kilgour et al., 2004; Sutto et al., 2004; Salathe, 2007; Weiterer et al., 2014; Perniss et al., 2020). Local milieu changes in pathophysiological conditions also influence mucociliary clearance, such as the formation of reactive oxygen species, and pathogenic bacterial, viral or fungal components mediate opposing effects and can reduce CBF with subsequent clinically relevant impairment in mucociliary clearance (Burman and Martin, 1986; Weiterer et al., 2015; Kamiya et al., 2020).

Some severe congenital diseases, such as primary cilia dyskinesia, are characterized by recurrent infections caused by a significant impairment of mucociliary clearance (Ren et al., 2023). However, acquired ciliary dysfunction is pivotal in many pathophysiologic conditions. For example, mucociliary clearance is impaired in tracheal intubated and mechanically ventilated patients, and mechanical ventilation is associated with increased bacterial colonization and pneumonia (Bassi et al., 2008). Furthermore, an overall impairment of mucociliary clearance was also found in critically ill patients in intensive care units (Nakagawa et al., 2005). Impairment of mucociliary clearance is already observed during general anesthesia with mechanical ventilation following endotracheal intubation and may even persist in the postoperative period (Adams et al., 2021). The temperature of the inspired air and the respirator settings modulate the degree of impairment, but the drugs used perioperatively also influence mucociliary clearance (Deng et al., 2023).

Some intravenous and volatile anesthetics, and perioperative antagonists, such as neostigmine and sugammadex, have been evaluated for their influence on mucociliary clearance (Forbes and Gamsu, 1979; Kesimci et al., 2008; Ozciftci et al., 2022). In addition, a recent experimental series showed that a drug commonly used to treat perioperative hypotension, a 20:1 mixture of cafedrine/theodrenaline, accelerated murine PTV and is, therefore, suggested to modulate mucociliary clearance when administered intravenously (Schmidt et al., 2023a). However, cafedrine/theodrenaline is solely used as an intravenous bolus applied for a short period to treat acute hypotensive states. When profound and prolonged hypotension persists, vasopressors, such as dopamine, norepinephrine, or vasopressin, are administered for longer periods in the perioperative setting and intensive care units (Ruiqiang et al., 2021; Douglas et al., 2023).

Dopamine, a precursor of norepinephrine that lacks one hydroxyl group, has been widely used to maintain adequate mean arterial pressures in different forms of shocks (Suzuki et al., 2022). Dopamine has different effects at different dosages. While there is a large inter-individual variability in the effects observed, low dosages induce vasodilation on visceral and renal vasculature via D_1_ receptors, and intermediate and high dosages act on β_1_- and α_1_-adrenergic receptors, leading to positive inotropy and vasoconstriction, respectively (Jozwiak, 2022; Ratnani et al., 2023). Patients who received dopamine after non-cardiac surgery had comparable mortality rates to those who received norepinephrine (Aoki et al., 2023). However, dopamine was associated with longer intensive care unit stays, and it led to a higher rate of cardiac arrhythmias and mortality in patients with septic shock than norepinephrine (Avni et al., 2015; Ruiqiang et al., 2021; Aoki et al., 2023). Therefore, norepinephrine has emerged as the first-line vasopressor recommended for patients with septic shock (Ruiqiang et al., 2021).

Norepinephrine is an endogenous catecholamine that is not only used in patients with septic shock but also in the perioperative setting when mean arterial pressure remains unstable after bolus injections, such as phenylephrine, ephedrine, or 20:1 cafedrine/theodrenaline (Kim et al., 2023). Norepinephrine primarily acts as an agonist of β_1_-and α_1_-adrenergic receptors, leading to increased inotropy and vasoconstriction, while the heart rate is mostly unaffected (Ruiqiang et al., 2021). Norepinephrine is currently the most frequently administered vasopressor to treat hypotensive states in various settings. Nevertheless, in patients with septic shock, adding vasopressin is suggested when inadequate mean arterial pressures persist despite higher norepinephrine doses (Ruiqiang et al., 2021).

Vasopressin is normally synthesized in the pituitary gland and acts on vasopressin receptors as an endogenous peptide hormone (Ruiqiang et al., 2021). While V_1_ receptors lead to vasoconstriction, renal V_2_ receptors have antidiuretic effects (Ammar et al., 2022). Vasopressin is used as a potent vasopressor in septic shock along with norepinephrine and other vasopressors to reduce the overall catecholamine dosages (Ruiqiang et al., 2021; Ammar et al., 2022). Furthermore, it is suggested to have beneficial effects on hypotensive states in patients with elevated pulmonary vascular resistance because it increases systemic vascular resistance but not pulmonary vascular resistance (Sarkar et al., 2015).

Since the impact of these three commonly used vasopressors on mucociliary clearance has not yet been investigated in detail, our experiments aimed to evaluate their influence on the PTV, which indicates mucociliary clearance in the lower airways, and reveal the specific signaling cascades through which they alter the PTV in the epithelium. Dopamine, norepinephrine, or vasopressin were applied to mouse tracheae, and the PTV was measured. Reverse transcription polymerase chain reactions (RT-PCR) were performed to assess the expression of vasopressin receptors in the murine trachea and isolated tracheal epithelium. Further experiments were conducted to determine the importance of calcium release, its origin, and the value of extracellular calcium entry.

## 2 Materials and methods

### 2.1 Drugs and buffer solutions

All preparations and experiments were performed in 4-(2-hydroxyethyl)-1-piperazine ethanesulfonic acid (HEPES) solution consisting of 10 mM HEPES, 5.6 mM KCl, 2.2 mM CaCl_2_, 11 mM glucose, 136 mM NaCl, and 2.2 mM MgCl_2_. NaOH was used to adjust the pH to 7.4 at 30°C. To enable experiments in Ca^2+^-free solutions, CaCl_2_ was omitted and 1 mM ethylene glycol-bis(β-aminoethyl ether)-N,N,N′,N′-tetraacetic acid was added. The following drugs were applied during the experiments: 2-aminoethoxydiphenylborane (2-APB; 40 µM diluted in 8 µL of dimethyl sulfoxide [DMSO]; TOCRIS Bioscience, Bristol, United Kingdom), adenosine triphosphate (ATP; 150 µM in 3 µL of H_2_O; Sigma-Aldrich, St. Louis, MO, United States), caffeine (30 mM in 2 mL of HEPES; Roth, Karlsruhe, Germany), CGP20712A (0.1 µM diluted in 2 µL of H_2_O or 100 µM diluted in 20 µL H_2_O; TOCRIS Bioscience, Bristol, United Kingdom), conivaptan (1 µM diluted in 2 µL of DMSO; Cayman Chemical, Ann Arbor, MI, United States), dopamine (0.58 µM in 100 µL of H_2_O; Fresenius Kabi GmbH, Bad Homburg, Germany), H-89 (10 µM diluted in 20 µL of DMSO; Sigma-Aldrich, St. Louis, MO, United States), nelivaptan (10 nM diluted in 2 µL of DMSO; Axon Medchem, Groningen, Netherlands), norepinephrine (1.21 µM in 100 µL of H_2_O; CHEPLAPHARM Arzneimittel GmbH, Greifswald, Germany), relcovaptan (10 µM diluted in 4 µL of DMSO; Cayman Chemical, Ann Arbor, MI, United States), SCH23390 (10 µM diluted in 2 µL of H_2_O; Enzo Life Sciences, Farmingdale, NY, United States), U-73122 (7.5 µM diluted in 4 µL of DMSO; Enzo Life Sciences, Farmingdale, NY, United States), and vasopressin (0.10 µM in 100 µL of H_2_O; US Biological Life Sciences, Swampscott, MA, United States). The reported drug concentrations were achieved during the experiments after applying the stock solution with the given volume to the 2 mL of HEPES buffer solution in the recording chamber.

### 2.2 Tracheal preparation and imaging

Male C57BL6J mice weighing 25–35 g (aged 12–15 weeks) were obtained from Charles River (Sulzfeld, Germany). All procedures involving animals were conducted in compliance with European legislation for the protection of animals used for scientific purposes and the standards for animal experiments according to the German animal welfare law. The experiments were approved by the local committee for animal care of the regional council (permit number: 851_M, regional council of Giessen, Germany).

After deep isoflurane (Baxter, Unterschleissheim, Germany) anesthesia, mice were sacrificed by intraperitoneal injection of pentobarbital (800 μg/kg). The following steps were performed immediately within 30 min after euthanasia, as previously described (Schmidt et al., 2023b). In brief, the trachea was dissected with a parasternal incision of the thorax and a median incision of the throat. It was then gently disconnected by slicing it cranial to its bifurcation and directly caudal to the larynx. The trachea was immediately transferred to a Delta T culture dish (Bioptechs, Butler, PA, United States) containing 2 mL of preheated HEPES buffer at pH 7.4°C and 30°C. The dish was pre-coated with Sylgard polymer (Dow Corning, Wiesbaden, Germany) to allow precise positioning of the trachea with two minutiae (Fiebig Lehrmittel, Berlin, Germany). The trachea was fixed so that the cartilage arches faced the Sylgard polymer and the pars membranacea, including the musculus trachealis, faced upward. During the preparations, connective tissues and surrounding blood vessels were gently resected using spring scissors (Vannas-Tübingen, FST, Heidelberg, Germany). Finally, preparation was completed when the musculus trachealis was cut open in a longitudinal direction, and the respiratory epithelium was directly visualized.

After the HEPES buffer was replaced, the trachea was transferred to the stage holder of an upright transmission light microscope (BX50 WI, Olympus, Hamburg, Germany). A temperature control unit maintained a constant temperature of 30°C in the center of the buffer solution, where the trachea was placed. According to the previously published methods, the optimal measuring conditions were realized at 30°C, although the PTV might be slightly slower than in real-time (Weiterer et al., 2015; Müller et al., 2021). Next, 3 µL of polymer particles (Dynabeads, Dynal Biotech GmbH, Hamburg, Germany) with a mean diameter of 2.8–4.5 µm were added to the buffer solution. The tracheal epithelium was then visualized between two cartilages in bright-field mode using a 20× water immersion lens (BW50 WI, Olympus, Hamburg, Germany), allowing the PTV to be measured by the controlled motion of the Dynabeads along the tracheal epithelium.

### 2.3 PTV measurement

The 80-min observation period began after a 30-min resting period, where repeated PTV measurements were made under the influence of the different drugs. The PTV was measured every 3 min during the first 72 min of the observation period. Next, ATP was applied to confirm the viability of the tracheal epithelium, leading to a maximal increase in PTV. Then, the PTV was measured every 2 min until the end of the experiment at 80 min. From these measurements, the average PTV was calculated for each timepoint, as previously described (Weiterer et al., 2014; Müller et al., 2021; Schmidt et al., 2023a).

### 2.4 RNA extraction from murine tissues.

The murine tracheae, tracheal epithelium, kidneys, and cerebrum were collected after euthanasia. The tracheal epithelium was isolated by gently scrubbing the epithelial layer from the opened trachea using a hygienic swab. All tissues were stored in an RNA stabilization solution (Invitrogen™ RNAlater™, Thermo Fisher Scientific, Waltham, MA, United States) at −20°C until further processing. Next, tissue samples were lysed in 350 µL RNeasy Lysis Buffer (Qiagen, Hilden, Germany) containing 3.5 µL of ß-mercaptoethanol and then homogenized in a tissue homogenizer (Precellys Evolution homogenizer, Bertin Technologies, Montigny-le-Bretonneux, France). RNA was extracted using the RNeasy Micro Kit, and DNA was removed using the RNase-Free DNase set Kit (both from Qiagen, Hilden, Germany).

### 2.5 RT-PCR

cDNA was synthesized using the QuantiTect Reverse Transcription Kit (Qiagen, Hilden, Germany) according to the manufacturer’s protocol and then stored at −20°C until further use. The expression of the arginine vasopressin receptors V_1_ (*Avpr1a*), V_1b_ (*Avpr1b*), and V_2_ (*Avpr2*) was analyzed in the whole trachea and the respiratory epithelium alone (n = 5 each) using specific primers and temperatures shown in Table 1. The primers were selected using the NCBI Primer designing tool (https://www.ncbi.nlm.nih.gov/tools/primer-blast, National Institutes of Health, Bethesda, MD, United States). The kidney served as the positive control for *Avpr1a* and *Avpr2*, while cerebrum was used as positive control for *Avpr1b*. The housekeeping gene RNA polymerase II subunit A (*Polr2a*) served as the control to ensure the quality of the RT-PCR and H_2_O was used as negative control. The primer concentration was set to 0.2 µM and RT-PCR was performed in a thermocycler (AlphaMetrix Biotech GmbH, Rödermark, Germany). The Taq polymerase (Qiagen, Hilden, Germany) was activated at 95° for 3 min, followed by 40 cycles consisting of a 1-min denaturation at 95°C, a 30-s annealing step at the primer-specific temperature (Table 1), and a 2-min extension at 72°C. PCR products were visualized using GelRed (Biotium, Fremont, CA, United States) with a digital imaging system (Vilber Lourmat, Eberhardzell, Germany) in agarose gel (1% an Tris-acetate-ethylenediaminetetraacetic acid buffer) after electrophoresis at 100 V for 60 min. The 100 bp GeneRuler (Thermo Fisher Scientific, Waltham, MA, United States) was used to verify the size of the PCR products.

**TABLE 1:** The RT-PCR primers, annealing temperatures, NCBI reference sequence, and product lengths for the murine vasopressin receptors and housekeeping genes.

Target	Gene	Sequence	NCBI reference sequence	Product length	Annealing temperature (°C)
V_1a_R	*Avpr1a*	Forward: 5′-TCTTCATCGTCCAGATGTGGTC-3′ Reverse: 5′-CCAGTAACGCCGTGATCGT-3′	NM_016847.2	90 bp	58.0
V_1b_R	*Avpr1b*	Forward: 5′-TCCAGGGCAAAGATCCGAAC-3′ Reverse: 5′- CAACAGGTGGCTGTTGAAGC-3′	NM_011924.2	222 bp	56.9
V_2_R	*Avpr2*	Forward: 5′- CTACCACGTCTGGCATTGCT-3′ Reverse: 5′- GCATGAGCAACACAAAGGGG-3′	NM_001276299.1	288 bp	58.0
POLR2a	*Polr2a*	Forward: 5′-GAGAAGCTGGTCCTTCGAATC-3′ Reverse: 5′-GCATGTTGGACTCAATGCATC-3′	NM_001291068.1	121 bp	58.0

### 2.6 Statistical analysis

Tracheal preparations and PTV measurements were only included in the statistical analyses when tracheal preparations showed directed particle motion during the whole observation period, and a clear response to the application of ATP was detected at the end of the experiments, immediately resulting in normalized PTV values of more than 150%, or a relative increase of more than +40% of the relative PTV. However, when high concentrations were applicated to obtain the concentration-response relationship, maximum PTV was already reached and could not be further accelerated by ATP. In this case, tracheal preparations were still included when directed particle motion was preserved during the whole observation period, and replicate experiments yielded similar results. The absolute PTV value was standardized to 100% after the resting time and before the observation period. The PTV values at distinct time points are presented as the mean and standard error of the mean (SEM). The peak plateau PTV values of each experimental group are presented as median and interquartile range (IQR). Half maximal effective concentrations (EC_50_) were calculated using the Hill equation, which is used to describe non-linear concentration-response relationships (Goutelle et al., 2008). All experimental groups for each analyzed substance were compared using a single two-way repeated measures analysis of variance model for each vasopressor, with inter-group differences over time evaluated using post-hoc Bonferroni tests. The data were statistically analyzed using the R statistical software, version 4.0.4 (www.r-project.org), and the figures were created using GraphPad PRISM (version 9.5.0, GraphPad Software, La Jolla, CA, United States). Two-tailed *p* -values of < 0.05 were generally considered statistically significant.

## 3 Results

### 3.1 Dopamine, norepinephrine, and vasopressin accelerate the PTV in a dose-dependent manner

**FIGURE 1:**
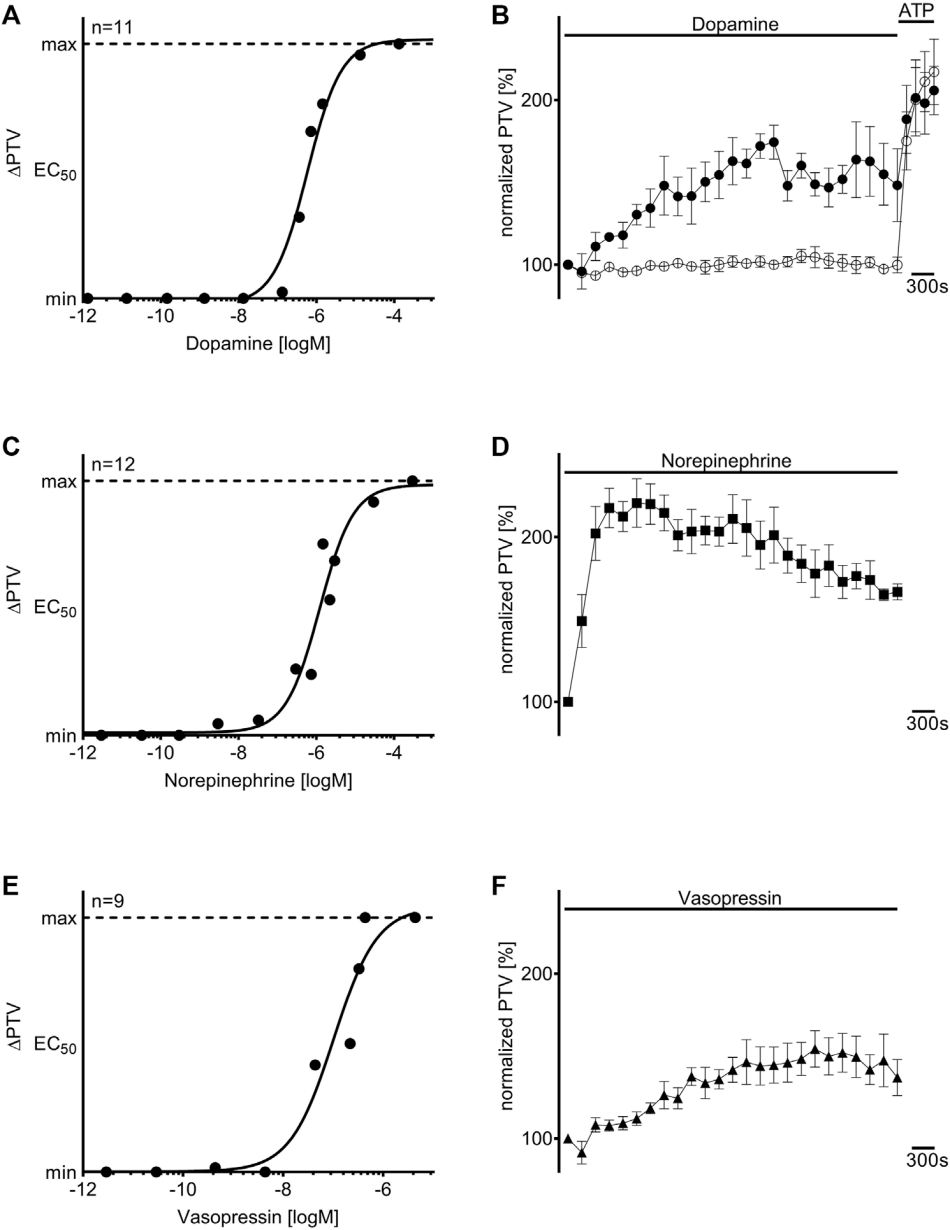
Dopamine, norepinephrine, and vasopressin increased the particle transport velocity (PTV) in a concentration-dependent manner. The Hill equation describes the concentration-response relationships for **(A)** dopamine, **(C)** norepinephrine, and **(E)** vasopressin. The “n” in **(A**,**C**,**E)** indicates the number of individual data points used for the calculation of the Hill equation, and each point (●) represents one tracheal preparation. Application of the calculated half maximal effective concentrations for **(B)** dopamine (●, 0.58 µM, n = 4), **(D)** norepinephrine (◼, 1.21 µM, n = 5), and **(F)** vasopressin (▲, 0.10 µM, n = 5) induced a long-lasting increase in the murine PTV; the PTV remained constant around its baseline value in control experiments (○, n = 5). ⊥ standard error of the mean (SEM).

When dopamine, norepinephrine, or vasopressin were applied to the murine tracheae, the PTV increased in a concentration-dependent manner. The concentration-response relationships were described using the Hill equation, revealing EC_50_ values of 0.58 µM for dopamine (n = 11), 1.21 µM for norepinephrine (n = 12), and 0.10 µM for vasopressin (n = 9, Figures 1A,C,E). Under control conditions, the PTV remained constant throughout the observation period (101 [97–106] %, n = 5). A steep increase in the PTV after administering ATP (150 µM) confirmed the vitality of the tissues at the end of each experiment, which is demonstrated by an example for all experiments in Figure 1B. When the calculated EC_50_ values were applied, the PTV significantly increased for the entire observation period, with the steepest increase observed with norepinephrine (227 [199–235] %, *p* < 0.001, n = 5, Figure 1D). The PTV plateaued following an initial flat rise after administering dopamine (179 [167–187] %, *p* < 0.001, n = 4, Figure 1B) and vasopressin (163 [146–177] %, *p* < 0.001, n = 5, Figure 1F). Subsequent experiments were conducted with the inhibitory substances to evaluate the value of substance-specific membrane-bound receptors. Since the initial kinetics and periods in the PTV increase differed among the applied substances, the inhibitors were incubated for 21 or 30 min before the application of dopamine/vasopressin or norepinephrine, respectively. Therefore, adequate observation periods after applying the three vasopressors were ensured throughout the experiments.

### 3.2 Dopamine, norepinephrine, and vasopressin increase the PTV via substance-specific receptors

**FIGURE 2:**
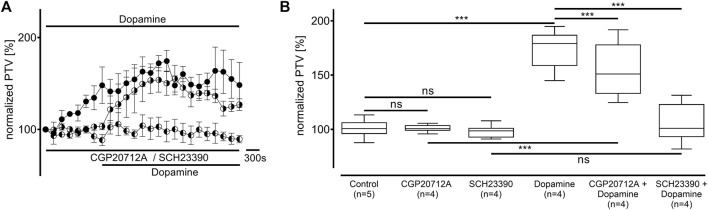
The increase in the PTV after administering dopamine depends on D_1_-like and, to a lesser extent, β-adrenergic receptor activation. **(A,B)** The PTV still increased when β-adrenergic receptors were inhibited with CGP20712A (100 µM). However, dopamine’s effects completely vanished when D_1_-like receptors were inhibited by the selective inhibitor SCH23390. Control curve for dopamine alone in **(A)** was taken from Figure 1B for better orientation. ***p < 0.001; ns: not significant; ● dopamine; ◐ dopamine + SCH23390 (10 µM); ◑ dopamine + CGP (100 µM); ⊥ standard error of the mean, box and whisker plots indicate the median, interquartile range (box), minimum, and maximum (whiskers).

Dopamine was administered to the tracheae in the presence of the β-adrenergic receptor inhibitor CGP20712A (100 μM, n = 4) and the dopamine D_1_/D_5_ receptor inhibitor SCH23390 (10 μM, n = 4), respectively. Both inhibitors did not alter the PTV compared to the control experiments (CGP20712A: 101 [99–104] %; SCH23390: 99 [94–116] %; Figure 2). The PTV significantly increased when dopamine was applied in the presence of CGP20712A (151 [139–166] %, *p* < 0.001, Figures 2A,B). The maximum PTV was significantly lower after dopamine was administered with than without β-adrenergic receptor inhibition (*p* < 0.001). However, no significant increase in the PTV was observed when D_1_-like receptors were inhibited, and the PTV remained comparable to control conditions (101 [94–116] %).

**FIGURE 3:**
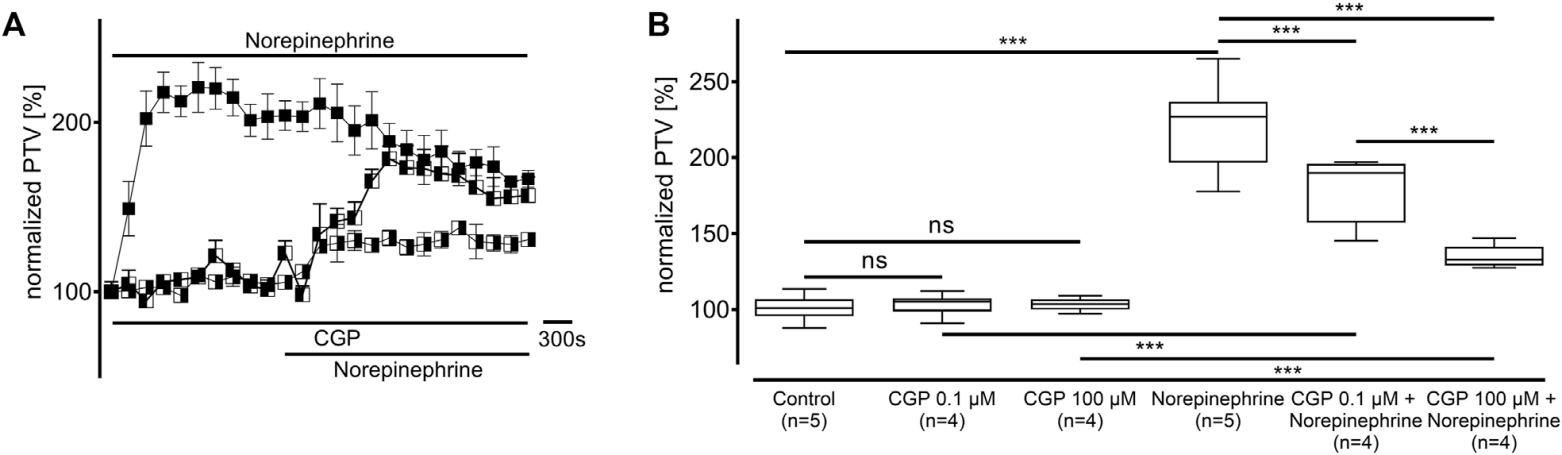
The change in the PTV after administering norepinephrine depends on β-adrenergic receptor activation. **(A)** Norepinephrine’s effect on the PTV was weaker when the adrenergic receptor blocker CGP20712A was applied at a β_1_-selective concentration (0.1 µM). The increase in the PTV after administering norepinephrine was further reduced when CGP20712A was applied at a higher, unselective β-adrenergic receptor inhibition concentration (100 µM). **(B)** CGP20712A alone did not alter the PTV. Control curve for norepinephrine alone in **(A)** was taken from Figure 1D for better orientation. ***p < 0.001, ns: not significant, ◼ norepinephrine, ◧ norepinephrine + CGP (0.1 µM), ◨ norepinephrine + CGP (100 µM), ⊥ standard error of the mean, box and whisker plots indicate median, interquartile range (box), minimum and maximum (whiskers).

Norepinephrine was applied in the presence of CGP20712A at a selective β_1_-adrenergic receptor inhibitory concentration (0.1 µM, n = 4) and an unselective β-adrenergic receptor inhibitory concentration (100 μM, n = 4, Figure 3). While both CGP20712A concentrations did not alter the PTV compared to the control experiments (0.1 µM: 105 [102–107] %; 100 µM: 104 [101–106] %), the PTV significantly increased in the presence of norepinephrine (0.1 µM: 190 [170–196] %, *p* < 0.001; 100 µM: 133 [129–141] %, *p* < 0.001; Figure 3A). However, compared to applying norepinephrine without CGP20712A, the maximum PTV was significantly lower with selective β_1_-adrenergic receptor inhibition (*p* < 0.001), and unselective β-adrenergic receptor inhibition (both *p* < 0.001, Figure 3B), with the latter showing a more significant decrease.

**FIGURE 4:**
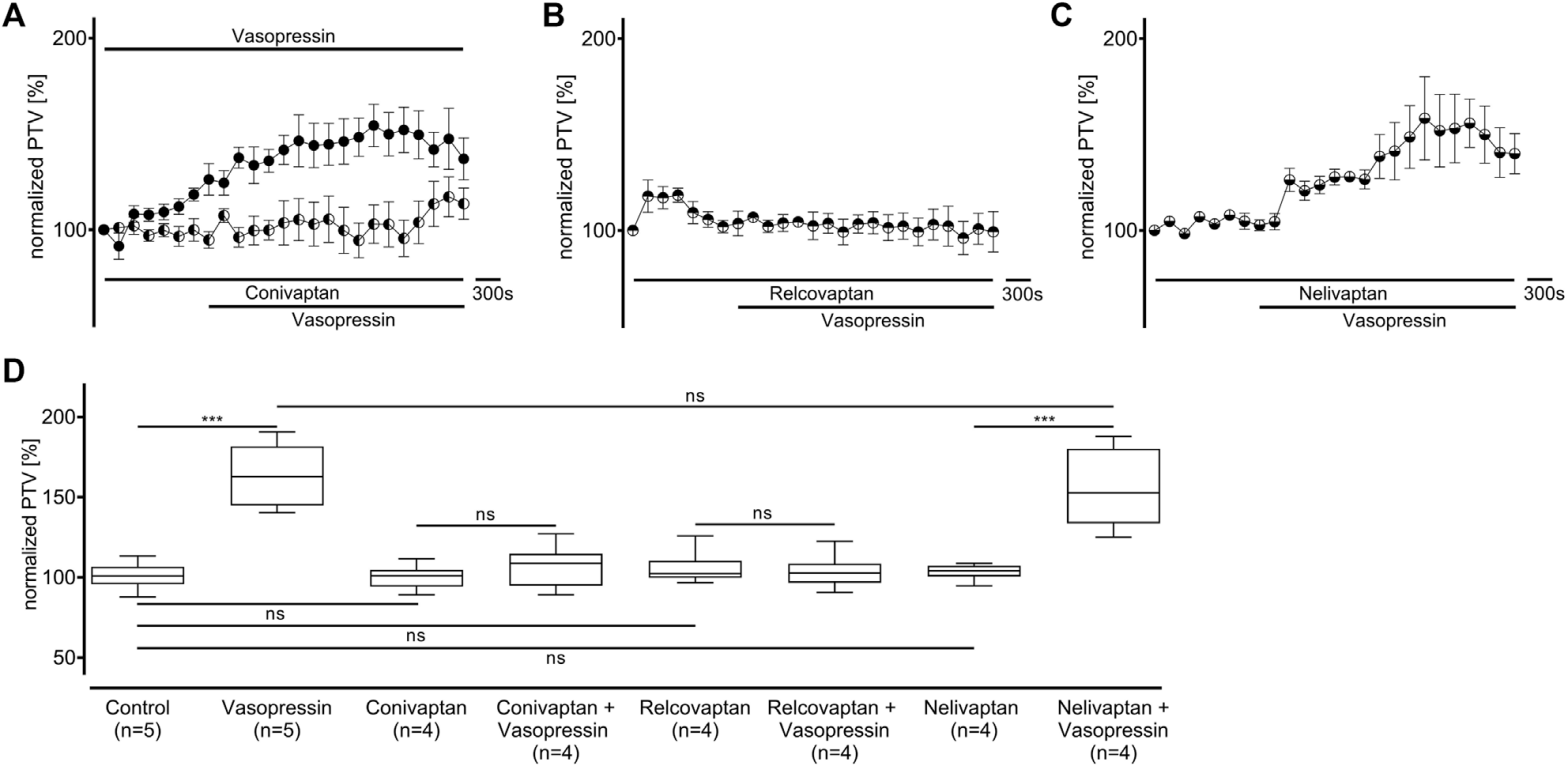
The increase in PTV after administering vasopressin depends on the V_1a_ receptor. **(A)** The vasopressin-dependent increase in the PTV completely vanished when the V_1a_ and V_2_ receptors were inhibited by conivaptan. **(B)** Vasopressin did not increase the PTV when the selective V_1a_ inhibitor relcovaptan was applied. **(C)** However, vasopressin increased the PTV when the V_1b_ receptors were inhibited. **(D)** Conivaptan, relcovaptan, and nelivaptan did not alter the PTV. Control curve for the vasopressin alone in **(A)** was taken from Figure 1F for better orientation ***p < 0.001, ns: not significant. ● vasopressin, ◐ vasopressin + conivaptan (1 µM), ◓ vasopressin + relcovaptan (10 µM), ◒ vasopressin + nelivaptan (10 nM), ⊥ standard error of the mean, box and whisker plots indicate median, interquartile range (box), minimum and maximum (whiskers).

**FIGURE 5:**
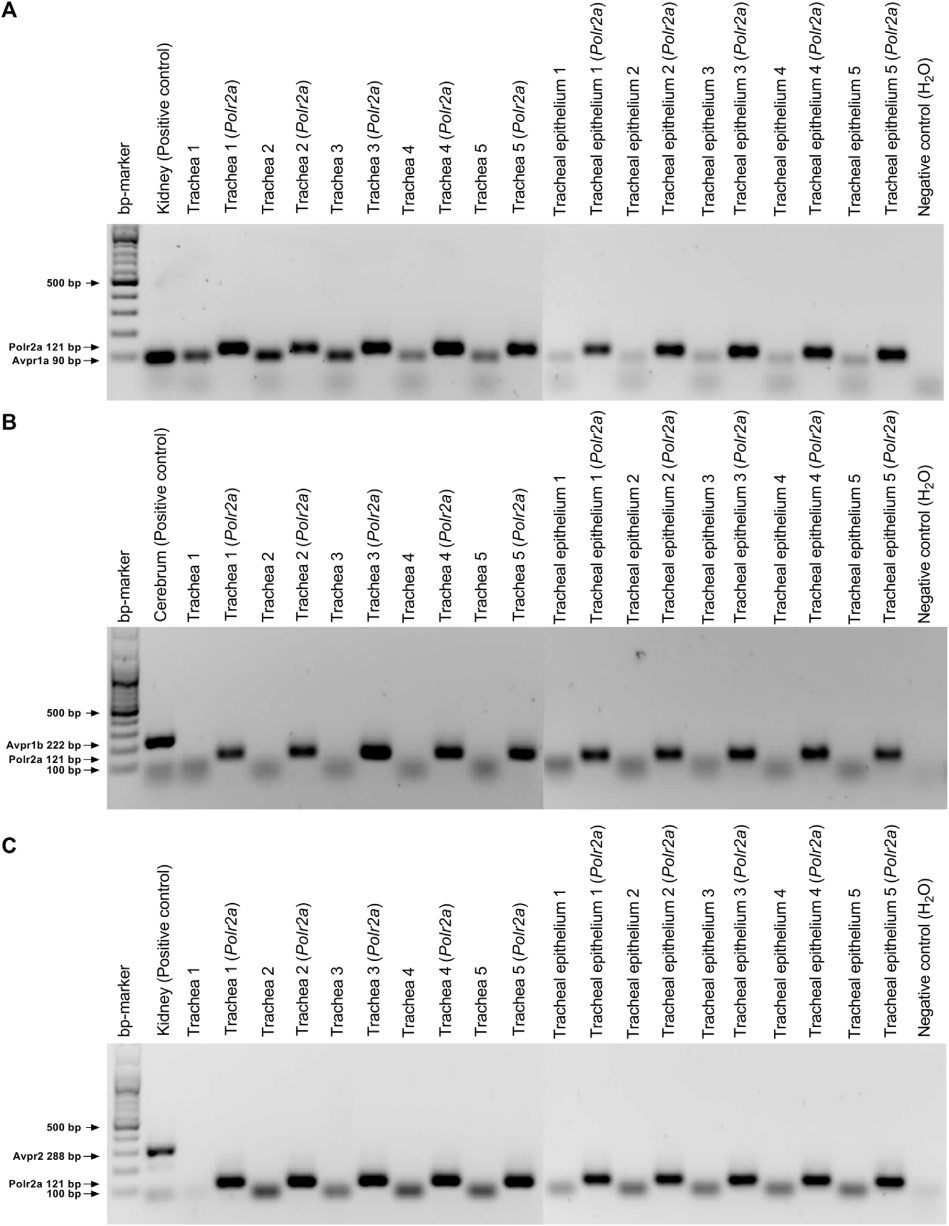
Expression analysis in mouse tissues using reverse transcriptase (RT)-PCR. **(A)** Vasopressin V_1a_ receptor (*Avpr*1a, 90 bp) was detected in murine trachea and tracheal epithelium, wherease transcripts encoding **(B)** V_1b_ (*Avpr*1b, 222 bp) and **(C)** V_2_ (*Avpr*2, 288 bp) were not detected. Kidney (*Avpr1a* and *Avpr2*) and cerebrum (*Avpr1b*) served as positive control for the vasopressin receptor primers. The housekeeping gene RNA polymerase II subunit A (*Polr2a,* 121 bp) and H_2_O were used as the control to ensure the quality of the RT-PCR and as negative control, respectively.

The experiments conducted on vasopressin receptors are shown in Figure 4. The value of the vasopressin receptors was initially evaluated in the presence of conivaptan (1 μM, n = 4), a selective V_1a_ and V_2_ receptor antagonist (Figure 4A). Conivaptan alone did not alter the basal PTV (101 [96–114] %). Moreover, the PTV did not significantly increase after vasopressin was applied (109 [96–114]). Subsequent experiments using the selective V_1a_ receptor antagonist relcovaptan (10 μM, n = 4) showed that the lack of increase in the PTV was entirely due to V_1a_ receptor inhibition. While relcovaptan alone did not change the PTV (102 [100–108] %), the PTV did not increase after applying vasopressin (103 [99–106] %; Figure 4B). Consequently, nelivaptan (10 nM, n = 4), a selective V_1b_ receptor antagonist, could not prevent the vasopressin-induced increase in the PTV (185 [169–189] %, *p* < 0.001), while the baseline PTV remained comparable in the presence of nelivaptan alone (101 [99–106], Figure 4C). The maximum PTV after applying vasopressin was comparable with and without V_1b_ receptor inhibition (Figure 4D). RT-PCR indicated the expression of *Avpr1a*, but not *Avpr1b and Avpr2* in both the whole trachea and the tracheal epithelium (Table 2). Native bands are shown in Figure 5.

**TABLE 2:** Vasopressin receptor mRNA expression.

	*Avpr1a*	*Avpr1b*	*Avpr2*
Positive control^a^	+	+	+
Whole trachea	+	−	−
Tracheal epithelium	+	−	−
Negative control (H_2_O)	−	−	−

^a^ Tissue used as positive control: kidney (*Avpr1a* and *Avpr2*) and cerebrum (*Avpr1b*).

The value of pivotal intracellular signal transduction enzymes was evaluated in the following experiments.

### 3.3 Influence of intracellular signal transduction enzymes

**FIGURE 6:**
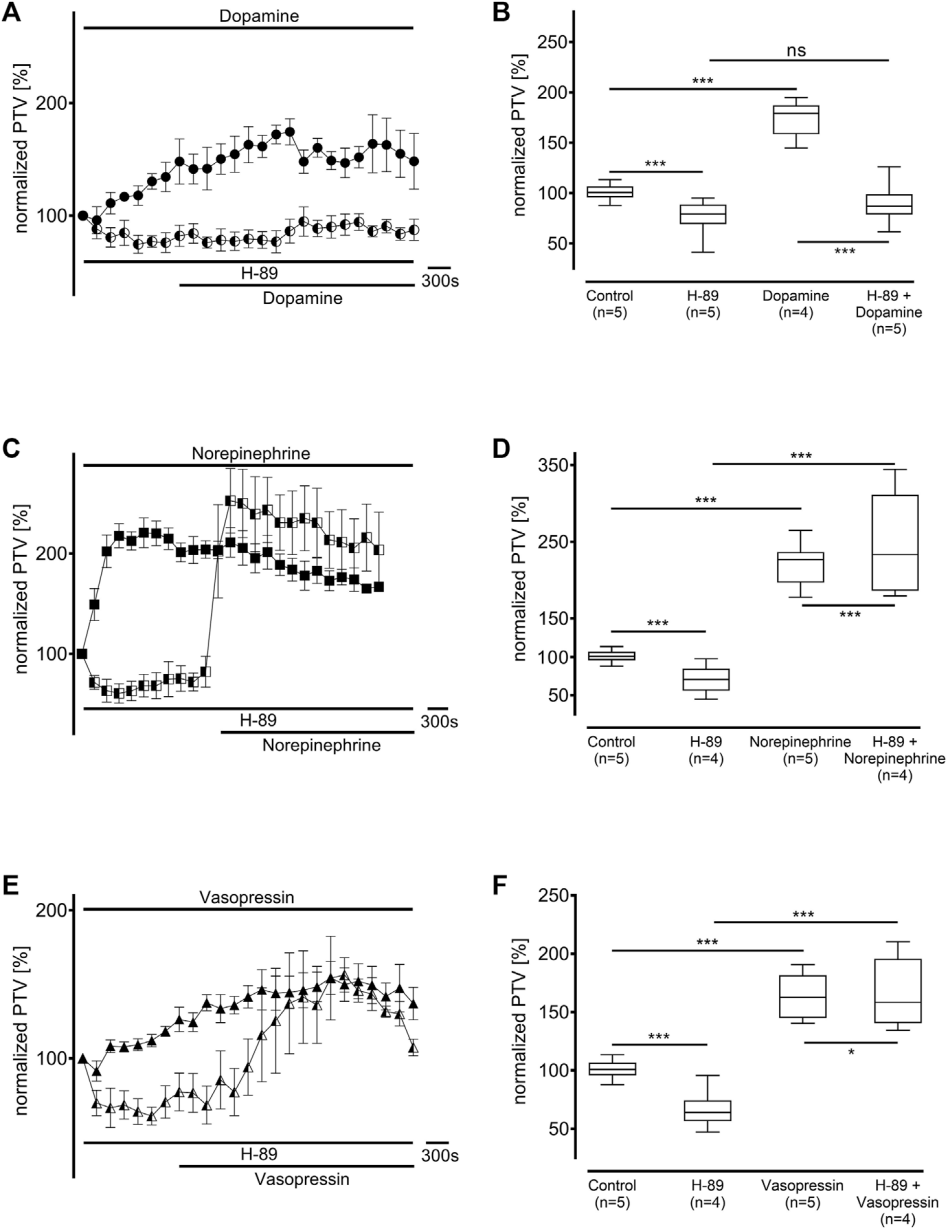
Protein kinase A (PKA) was inhibited by H-89 (10 µM). The baseline PTV decreased when the selective inhibitor was applied. **(A,B)** The PTV did not increase when dopamine was applied in the presence of H-89. However, **(C,D)** norepinephrine and **(E,F)** vasopressin significantly increased the PTV. Control curves for the agonists alone in **(A)**, **(C),** and **(E)** were taken from Figures 1B,D,F, respectively, for better orientation. *p < 0.05, ***p < 0.001, ns: not significant. ● dopamine, ◐ dopamine + H-89 (10 µM), ◼ norepinephrine, ◧ norepinephrine + H-89 10 μM, ▲ vasopressin, ◭ vasopressin + H-89 10 μM, ⊥ standard error of the mean, box and whisker plots indicate median, interquartile range (box), minimum and maximum (whiskers).

**FIGURE 7:**
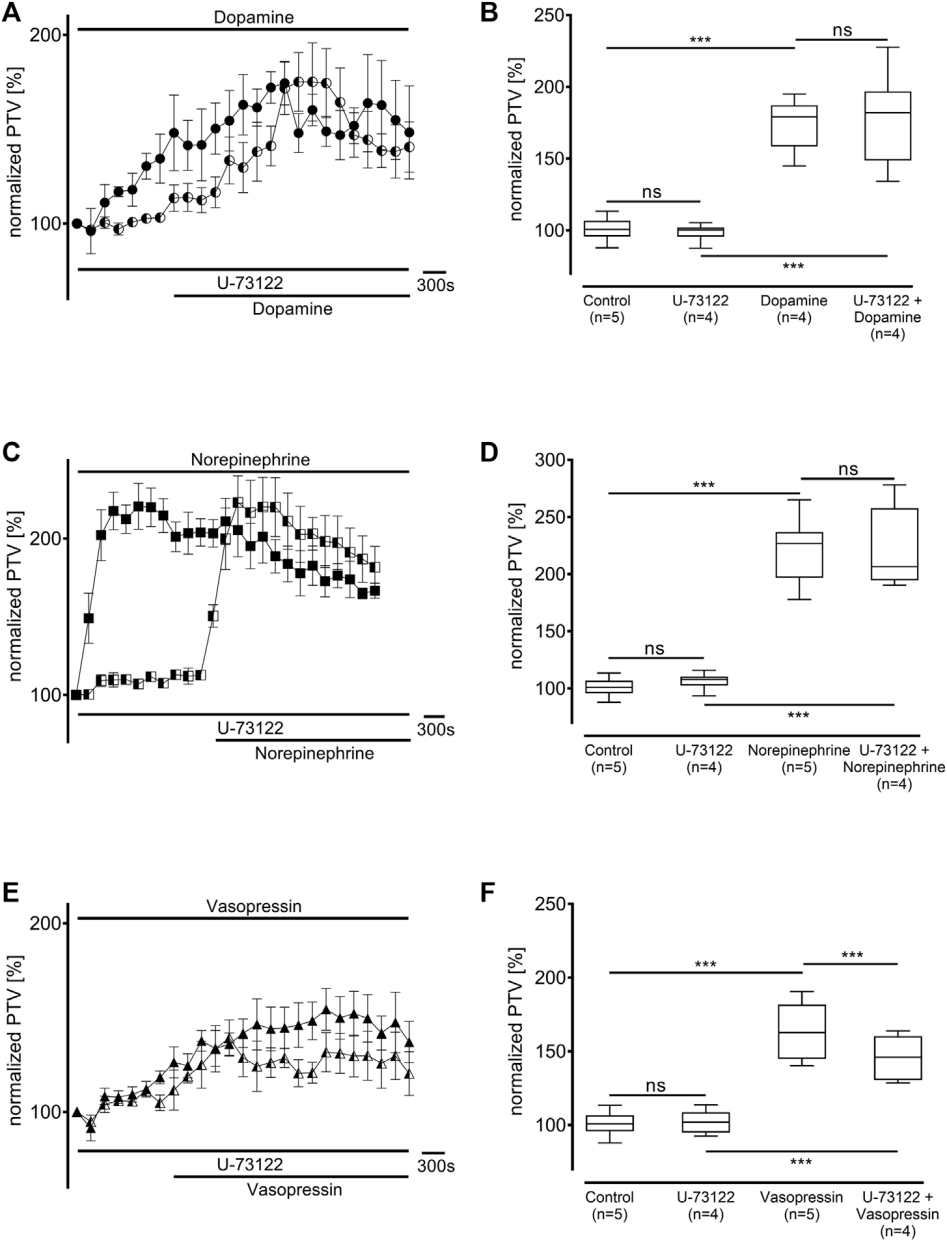
The increase in PTV with dopamine and norepinephrine but not vasopressin was independent of phospholipase C (PLC). The selective PLC inhibitor U-73122 did not alter the baseline PTV. **(A,B)** Dopamine and **(C,D)** norepinephrine still induced an equivalent increase in the PTV. **(E,F)** The PTV plateau was significantly reduced after the administration of vasopressin. Control curves for the agonists alone in **(A)**, **(C)**, and **(E)** were taken from Figures 1B,D,F, respectively, for better orientation. ***p < 0.001, ns: not significant. ● dopamine, ◐ dopamine + U-73122 (7.5 µM), ◼ norepinephrine, ◧ norepinephrine + U-73122 (7.5 µM), ▲ vasopressin, ◭ vasopressin + U-73122 (7.5 µM), ⊥ standard error of the mean, box and whisker plots indicate median, interquartile range (box), minimum and maximum (whiskers).

Protein kinase A (PKA) was inhibited using H-89 (10 µM), which generally significantly reduced the baseline PTV (dopamine: 79 [70–88] %, n = 5; norepinephrine: 70 [61–78] %, n = 4; vasopressin: 64 [58–70] %, n = 5: all *p* < 0.001, Figure 6). While the PTV did not significantly increase after applying dopamine (87 [81–99] %, Figures 6A,B), the PTV did significantly increase after applying norepinephrine and vasopressin despite the presence of H-89 (norepinephrine: 233 [180–270] %, *p* < 0.001, vasopressin: 158 [143–179] %, *p* < 0.001, Figures 6C–F). Subsequent experiments examined phospholipase C (PLC, each n = 4) inhibition using the selective inhibitor U-73122 (7.5 µM). U-73122 alone did not alter the PTV (dopamine: 100 [98–102] %; norepinephrine: 108 [103–110] %; vasopressin: 102 [95–109] %, Figure 7). However, the PTV significantly increased after applying dopamine, norepinephrine, and vasopressin (dopamine: 182 [162–194] %, *p* < 0.001, norepinephrine: 207 [201–226] %, *p* < 0.001, vasopressin: 146 [132–160] %, *p* < 0.001). After applying dopamine and norepinephrine, the maximum PTV was comparable with and without PLC inhibition (Figures 7A–D). However, after applying vasopressin, the maximum PTV was significantly lower with than without PLC inhibition (*p* < 0.001, Figures 7E,F). The following experiments evaluated the importance of extracellular and intracellular Ca^2+^ release for the increase in PTV.

### 3.4 Influence of intracellular and extracellular Ca^2+^ stores

**FIGURE 8:**
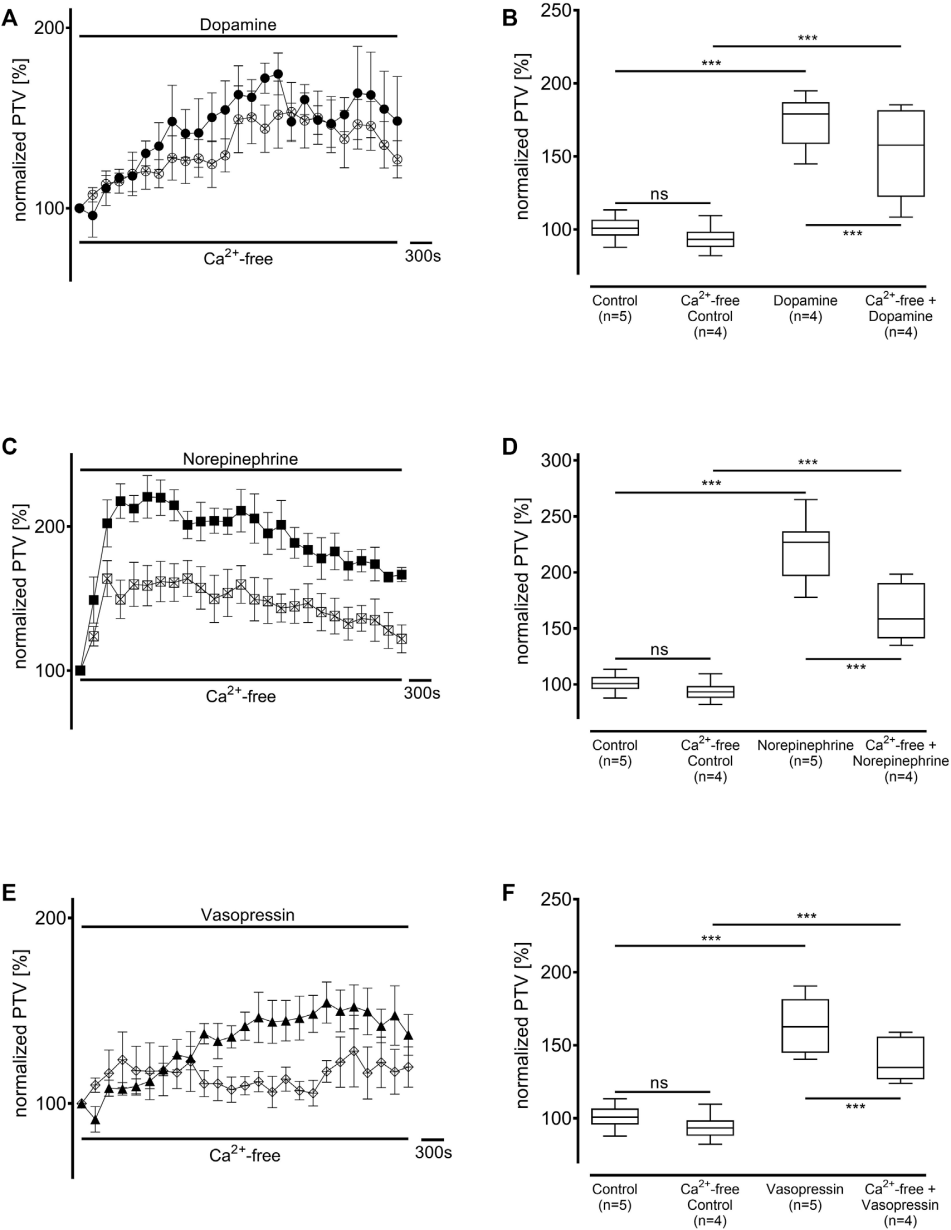
The full PTV increase is only realized with extracellular Ca^2+^ entry. The PTV was measured in a Ca^2+^-free buffer solution after applying **(A,B)** dopamine, **(B,C)** norepinephrine, and **(E,F)** vasopressin. Control curves for the agonists alone in **(A)**, **(C)**, and **(E)** were taken from Figures 1B,D,F, respectively, for better orientation. *p < 0.05, ***p < 0.001, ns: not significant. ● dopamine, ○ dopamine in Ca^2+^-free buffer, ◼ norepinephrine, □ norepinephrine in Ca^2+^-free buffer, ▲ vasopressin, ◇ vasopressin in Ca^2+^-free buffer, ⊥ standard error of the mean, box and whisker plots indicate median, interquartile range (box), minimum and maximum (whiskers).

Ca^2+^-free buffer solution was used to prevent extracellular Ca^2+^ entry after administering dopamine, norepinephrine, or vasopressin (Figure 8, each n = 4). The baseline PTV did not differ significantly between the Ca^2+^-free buffer solution and the Ca^2+^-containing buffer solutions (93 [90–98] %). Dopamine, norepinephrine, or vasopressin all induced a significant increase in the PTV in the Ca^2+^-free buffer solution, which followed comparable kinetics to the increases in Ca^2+^-containing buffer solutions (dopamine: 158 [136–182] %, *p* < 0.001, norepinephrine: 158 [146–177] %, *p* < 0.001, vasopressin: 135 [128–156] %, *p* < 0.001, Figures 8A,C,E). However, the maximum PTV was significantly lower in Ca^2+^-free than in Ca^2+^-containing buffer solutions after applying dopamine, norepinephrine, or vasopressin (all *p* < 0.001, Figures 8B,D,F).

**FIGURE 9:**
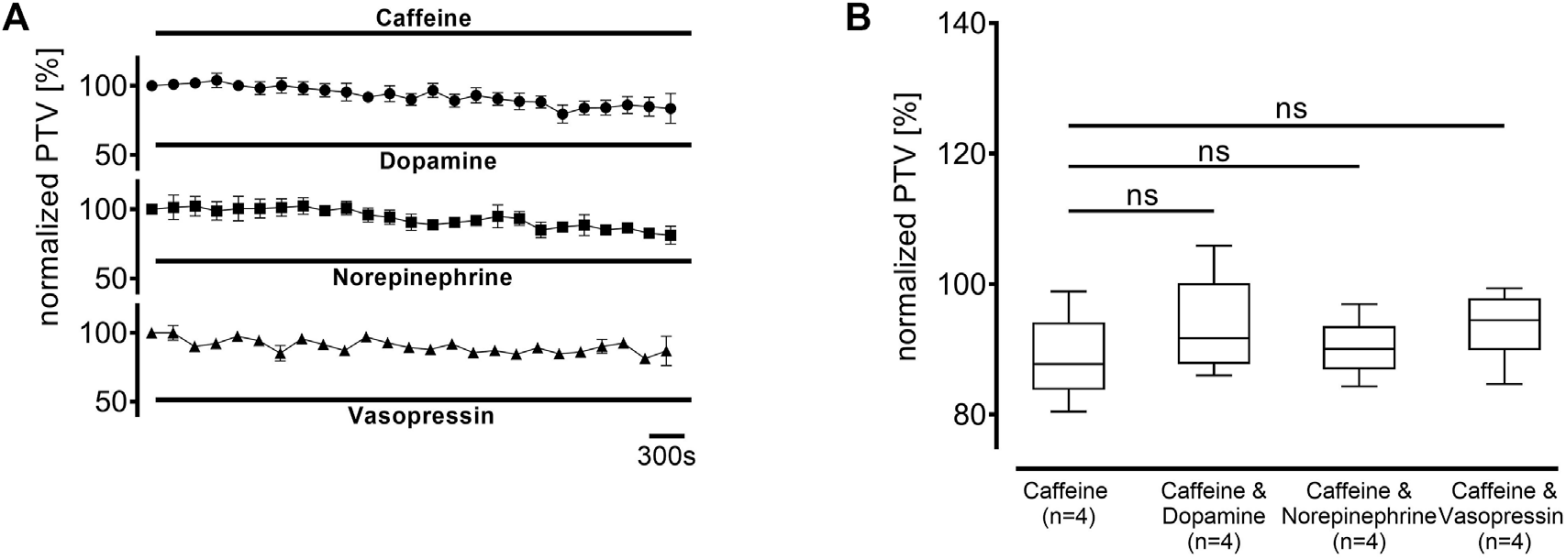
The PTV increase depends entirely on the depletion of intracellular Ca^2+^ stores. Intracellular Ca^2+^ stores were depleted using caffeine 30 min prior to the observation period. When **(A)** dopamine, norepinephrine, or vasopressin were applied, **(B)** no increase was observed. ns: not significant. ○ caffeine 30 mM, ● dopamine + caffeine 30 mM, ◼ norepinephrine + caffeine 30 mM, ▲ vasopressin + caffeine 30 mM, ⊥ standard error of the mean, box and whisker plots indicate median, interquartile range (box), minimum and maximum (whiskers).

Since the increase in the PTV was not entirely abolished by preventing extracellular Ca^2+^ entry, the next experiments aimed to examine the origin of intracellular Ca^2+^. Therefore, caffeine-sensitive Ca^2+^ stores, which are foremost the endoplasmic reticulum (ER), were depleted by caffeine (30 mM, each n = 4) in a Ca^2+^-free buffer solution at the beginning of the resting period. A transient increase in PTV through Ca2+ release following store depletion returned to a stable plateau prior to normalization at the beginning of the observation period. Dopamine, norepinephrine, and vasopressin did not induce any increase in the PTV compared to caffeine alone (caffeine alone: 88 [85–94] %, dopamine: 92 [89–96] %; norepinephrine: 90 [87–93] %; vasopressin: 94 [90–98] %, Figure 9).

**FIGURE 10:**
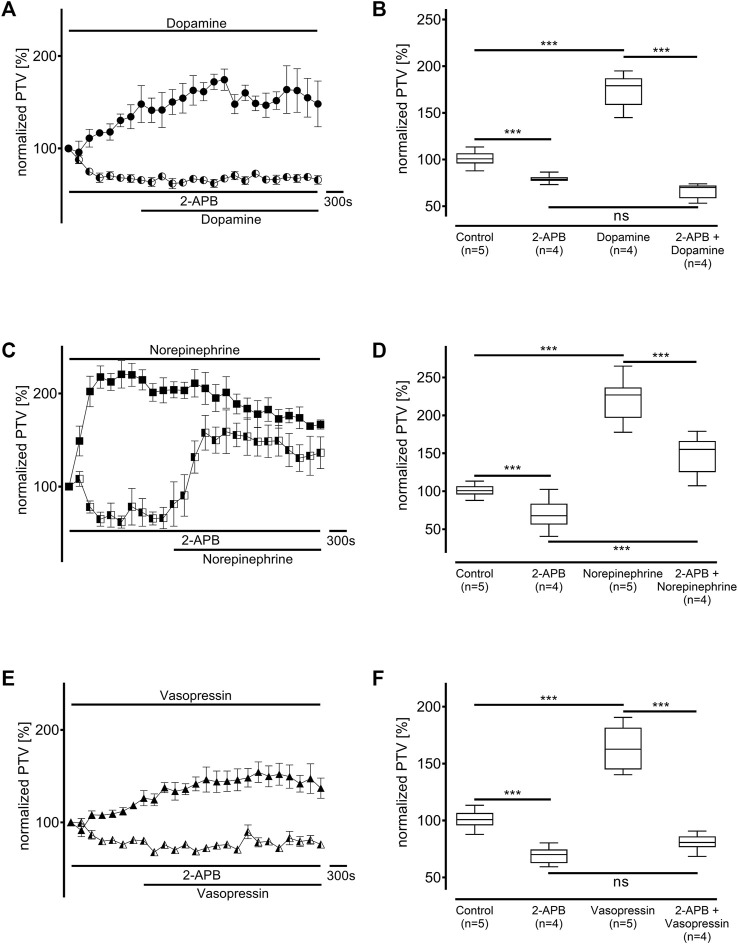
Inositol trisphosphate (IP_3_) receptor activation was pivotal to the increase in the PTV after administering dopamine and vasopressin. The IP_3_ receptors were selectively inhibited by 2-aminoethoxydiphenylborane (2-APB). When **(A,B)** dopamine or **(E,F)** vasopressin were applied, no increase in the PTV was observed compared to the controls. **(C,D)** However, the PTV significantly increased after applying norepinephrine. Control curves for the agonists alone in **(A)**, **(C)**, and **(E)** were taken from Figures 1B,D,F, respectively, for better orientation. ***p < 0.001, ns: not significant. ● dopamine, ◐ dopamine + 2-APB (40 µM), ◼ norepinephrine, ◧ norepinephrine + 2-APB (40 µM), ▲ vasopressin, ◭ vasopressin + 2-APB (40 µM), ⊥ standard error of the mean, box and whisker plots indicate median, interquartile range (box), minimum and maximum (whiskers).

Next, the inositol trisphosphate (IP_3_) receptors were inhibited with 2-APB (40 μM, each n = 4) to determine the value of IP_3_ receptor-associated Ca^2+^ release from the ER. Their inhibition significantly reduced the baseline PTV (dopamine: 79 [78–81] %, *p* < 0.001, norepinephrine: 68 [63–80] %, *p* < 0.001, vasopressin: 70 [63–74] %, *p* < 0.001, Figure 10). The PTV did not increase after applying dopamine (70 [61–71] %, Figures 10A,B). However, the PTV did significantly increase after applying norepinephrine (155 [140–165] %, *p* < 0.001), although the maximum plateau was significantly lower with than without IP_3_ receptor inhibition (*p* < 0.001, Figures 10C,D). Notably, the increase in the PTV was completely preserved when vasopressin was applied (81 [78–85] %; Figures 10E,F).

## 4 Discussion

Our study revealed that dopamine, norepinephrine, and vasopressin accelerate the murine PTV, suggesting changes in mucociliary clearance of the respiratory epithelium. All three analyzed substances showed concentration-response relationships, which can be described with the Hill equation, indicating specific receptor-mediated effects. Each substance ultimately leads to intracellular Ca^2+^ release, mediating the increase in the PTV, intensified by additional extracellular Ca^2+^ entry. However, dopamine, norepinephrine, and vasopressin use different, substance specific membrane-bound receptors to induce these effects.

Our experiments revealed that dopamine increases the murine PTV via the D_1_/D_5_ receptors, which are frequently referred to D_1_-like receptors (Montalant et al., 2023). These G protein-coupled receptors induce the formation of cAMP, which ultimately activates the PKA (Bozzi and Borrelli, 2013). Our experiments suggested that PKA is the pivotal enzyme in the signal transduction cascade that increases the PTV after dopamine administration. Therefore, our results are consistent with the known signal transduction cascade after D_1_-like receptor activation (Montalant et al., 2023). Ca^2+^ release was ultimately triggered by IP_3_ receptor activation, which was shown by the complete inhibition of any increase in the PTV after IP_3_ receptor inhibition. IP_3_ receptors are generally regulated by both cAMP- and PKA-dependent pathways (Taylor, 2017). PKA phosphorylates IP_3_ receptors after increasing cAMP concentrations, causing rapid, transient, and reversible changes in IP_3_ receptor channel function (Betzenhauser and Yule, 2010). While phosphorylation does not directly open IP_3_ receptors, phosphorylated IP_3_ receptors showed an increased open probability when activated by IP_3_, and closed receptor states were destabilized (Taylor, 2017). Therefore, PKA-induced phosphorylation can approximately double IP_3_ receptor-mediated Ca^2+^ release (Nakade et al., 1994). While closely related, each subtype has different phosphorylation sites, resulting in only the activity of subtypes 1 and 2 but not subtype 3 being enhanced by PKA-induced phosphorylation (Nakade et al., 1994; Taylor, 2017). Moreover, cAMP has also been shown to directly affect IP_3_ receptors without the concomitant activation of PKA (Taylor, 2017). However, these effects were primarily described in combination with parathyroid hormone, and it remains unclear whether they might also apply to other substances and especially to respiratory tissues (Tovey et al., 2008; Tovey et al., 2010).

Our experiments also suggested that dopamine affects β-adrenergic receptors to a lesser extent, proven by the PKA not being the pivotal receptor enzyme with norepinephrine in our experiments, and other β-adrenergic agonists in recent studies (Schmidt et al., 2023a). However, it must be noted that signal transduction after dopamine administration was only examined with one concentration reflecting the calculated EC_50_. Because dopamine generally showed concentration-response kinetics according to the Hill equation, it appears that D_1_-like receptors remain the pivotal receptor triggering increase in the PTV, with other adrenergic receptors barely affected. Therefore, only β-adrenergic receptors appear relevant, because no PTV-altering effect was found after α-receptor activation, and RT-PCR only detected the expression of the α_1_D subunit in murine respiratory epithelium in other studies (Weiterer et al., 2015).

Norepinephrine induced a steeper increase in the PTV than dopamine and vasopressin. Its observed kinetics were comparable with other β-adrenergic receptor agonists, such as theodrenaline, which has been recently examined (Schmidt et al., 2023b). We observed that norepinephrine alters the PTV via unselective β-adrenergic receptor stimulation because the PTV was further reduced when the concentration of CGP20712A was increased from selective β_1_-adrenergic inhibition to unselective β-adrenergic receptor inhibition. However, even the high concentration could not entirely prevent the increase in the PTV, although PTV was considerably reduced.

Therefore, we conclude that norepinephrine might increase the PTV via unselective β-adrenergic receptor activation since it is already known that PTV increases are independent of α-adrenergic receptor activation (Weiterer et al., 2015). Furthermore, our experiments showed independence from PLC, strengthening this finding because PLC is the main effector enzyme of the α_1_-adrenergic receptor. Nevertheless, since the slope of the concentration-response curve for norepinephrine was very steep, it seems possible that the calculated EC_50_ represents a slightly right-shifted position on the actual concentration-response curve. Therefore, the inhibitory concentration used in our experimental setup may not be sufficient to block a potentially higher norepinephrine concentration. Interestingly, PKA was not the pivotal signal transduction enzyme in the increase in the PTV after β-adrenergic receptor stimulation. This finding has also been observed after applying cafedrine and theodrenaline, which are both regarded as selective β_1_ receptor agonists (Schmidt et al., 2023b). Notably, the effects of these β_1_ receptor agonists were ultimately mediated via IP_3_ receptors which was not observed in our experiments with norepinephrine. Therefore, IP_3_ receptors might contribute to Ca^2+^ release because the maximum PTV was slower with than without IP_3_ receptor inhibition. Different signal transduction mechanisms and receptor involvement could also be hypothesized. The linkage between β-adrenergic receptors and ryanodine receptor activation releasing Ca^2+^ from the ER or sarcoplasmic reticulum in cardiomyocytes is generally well known (Zhou et al., 2009; Berisha et al., 2021). Ryanodine receptors 2 and 3 were found in the murine respiratory epithelium (Schmidt et al., 2023b). However, allosteric modulation of ryanodine receptors by cAMP, which could lead to Ca^2+^ release and the subsequent increase in the PTV despite its independence from PKA, has only been described for ryanodine receptor 1 (Cholak et al., 2023). Therefore, further studies should be performed with other β-agonists, which might confirm our findings, and inhibitory substances, which could elucidate the distinct signal transduction cascades.

Our experiments suggested that vasopressin accelerated the murine PTV via IP_3_ receptor triggered Ca^2+^ release from the ER. The relevance of extracellular Ca^2+^ entry after vasopressin administration was underscored by the markedly reduced PTV in the experiments using a Ca^2+^-free buffer solution. While the experiments with H-89 showed that PKA does not influence the change in PTV after vasopressin administration, the experiments with U-73122 suggested that PLC significantly influenced the change. These results are consistent with the typical V_1_ receptor signal transduction cascade because these G-coupled receptors activate PLC and ultimately release Ca^2+^ from the ER via IP_3_ receptor activation (Birnbaumer, 2000). In contrast, PKA is the main effector enzyme of V_2_ receptors, whose relevance would have been suggested if the PTV had been modulated by PKA inhibition (Birnbaumer, 2000). Our experiment with the nonspecific V_1a_ and V_2_ receptor inhibitor conivaptan initially suggested the relevance of one of these receptors. However, the increase in the PTV entirely disappearing when the V_1a_ receptors were selectively inhibited precluded further experiments with selective V_2_ inhibitors. Furthermore, V_1b_ receptor inhibition did not prevent the increase in the PTV after vasopressin administration, and subsequent RT-PCR only detected the expression of V_1a_ in the murine trachea and tracheal epithelium. Further experiments including immunohistochemistry could focus on the detailed characterization and localization of vasopressin receptors in the murine trachea and tracheal epithelium. While the presence of vasopressin receptors in mammalian lung tissues is generally accepted, their physiological function has not been widely examined (Tahara et al., 1998; Bernard et al., 2005). Vasopressin has been shown to regulate chloride secretion in human bronchial epithelial cells, suggesting that it can complexly regulate mucociliary clearance function not only by increasing the active outward transportation processes but also mucus formation (Bernard et al., 2005). Consistent with our results, V_1_ receptors also stimulated mucus production. Furthermore, even V_2_ receptors were highly relevant depending on the chloride concentration gradients between the mucosal and serosal sides of the epithelial cells (Bernard et al., 2005). Tamaoki et al. found that vasopressin increased the CBF of rabbit tracheal epithelium via Ca^2+^ release by thapsiargin-sensitive stores (Tamaoki et al., 1998). Since this study only measured CBF, its authors speculated that the observed effect of vasopressin might be translated into enhanced mucociliary transport, which our experiments have now confirmed (Tamaoki et al., 1998).

Our experiments have increased the knowledge about the roles of PLC and IP_3_ receptors in releasing intracellular Ca^2+^ after vasopressin receptor stimulation in murine tracheal tissues. Interestingly, some of our findings show important differences from those of Tamaoki et al. and must be discussed (Tamaoki et al., 1998). Firstly, Tamaoki et al. found no significant difference in the rabbit CBF increase when extracellular Ca^2+^ was depleted. In contrast, our experiments showed an impressive reduction in the murine PTV following the application of vasopressin in a Ca^2+^-free buffer solution. Secondly, while the relevance of the V_1_ receptor pathway is unquestionable in both studies, their results differ regarding whether the V_1a_ or V_1b_ receptor is the main receptor responsible for the observed effects.

When the chloride secretion ability of vasopressin receptors was examined in human bronchial epithelial cell lines, vasopressin’s effects were completely inhibited when both receptor subtypes were separately inhibited by highly specific inhibitors, suggesting the involvement of a V_1_-like receptor (Bernard et al., 2005). However, both V_1a_ and V_1b_ receptors were detected in subsequent RT-PCR (Bernard et al., 2005). Consistent with our experiments, Tamaoki et al. demonstrated V_1_ receptor involvement in the rabbit CBF by its increase disappearing after applying OPC-21268, an unselective V_1_ receptor inhibitor (Tamaoki et al., 1998). However, when they measured intracellular Ca^2+^ concentrations after administering selective V_1a_ or V_1b_ agonists, they increased more with the V_1b_ agonist and were not significantly reduced by the selective V_1a_ inhibitor CTM-vasopressin (Bernard et al., 2005). Whether these inconsistent results reflect differences between mammalians or different tissues, such as tracheae and bronchial epithelium, should be carefully evaluated in further studies where respiratory tissues from the same location should be compared with the same inhibitory substances under comparable experimental settings.

It must also be noted that in clinical practice, vasopressin is mainly administered with norepinephrine to treat vasodilatory shocks. Our experiments examined both substances separately to elucidate their substance-specific signal transduction pathways. However, when co-administered, their additive effects on the PTV appear conclusive at the respiratory epithelium because norepinephrine and vasopressin both target highly specific receptors and ultimately lead to Ca^2+^ release from the ER via different, substance-specific mechanisms.

All three vasopressors examined in our experiments showed a significantly reduced maximum PTV when administered in a Ca^2+^-free buffer solution. The maximum PTV was also significantly reduced with dopamine in a Ca^2+^-free buffer solution, but the numerical decline was low, questioning the clinical relevance of that finding. The PTV amplitude markedly declined with norepinephrine and vasopressin, suggesting greater relevance. Furthermore, no increase in the PTV was observed after administering the three substances when intracellular caffeine sensitive Ca^2+^ stores were depleted. Therefore, we conclude that norepinephrine, vasopressin, and, to a lesser extent, dopamine use extracellular Ca^2+^ influx to ultimately affect the PTV.

Extracellular Ca^2+^ influx occurs via store-operated Ca^2+^ entry (SOCE), which is triggered by the release of Ca^2+^ from intracellular stores (Spinelli and Trebak, 2016). SOCE is modulated by different receptors and channels, such as the ORAI calcium release-activated calcium channels (Nguyen et al., 2018). The ORAI channels are activated by stromal interacting molecules located in the transmembrane area of the ER that sense decreased Ca^2+^ concentration in the ER (Umemura et al., 2023). Future studies should conduct further experiments evaluating the value of SOCE in tracheal epithelial cells, which could use isolated tracheal epithelial cells with calcium fluorescence measurements. While specific inhibitory substances of SOCE should be used, specific inhibition of distinct channels is difficult in the face of multiple potential targets and complex interactions (Spinelli and Trebak, 2016). Therefore, we decided to prevent overall extracellular Ca^2+^ entry using a Ca^2+^-free buffer solution, and these results should be considered hypothesis-generating.

Our study had the following limitations that must be acknowledged. Firstly, our experiments used murine tissues. Therefore, their results should be generalized cautiously to human tissues and patients. Using murine tissues in our experiments is a generally valid approach to evaluate hypotheses that can later be proven in experiments with human tissues. However, some differences might be significant even between different mammalians. For example, while the V_1b_ receptor seems to provoke the increase in the CBF in rabbit tissues, the V_1a_ receptor seems to cause these effects in murine tissues. Secondly, isolated tracheae were used in all experiments where subsequent intravenous drug administration was impossible, with the drugs administered directly into the buffer solution in which the tracheae were placed. However, the physiological integrity of the tracheae was preserved, and the epithelial barrier might have prevented drug entry from the apical side. Therefore, the concentration-response relationship might vary considerably in other tissues and clinical practice. Thirdly, the clearance function of tracheae might differ from the lower respiratory tracts, which primarily contribute to mucociliary clearance. Fourthly, the intravenous concentrations of the examined vasopressors are unknown in clinical practice and might even have significant interindividual differences, limiting the transferability of the concentrations evaluated in our experiments. Concentrations might also vary considerably between different tissues at different time points. For example, the examined vasopressors are mainly administered intravenously via central venous lines, although norepinephrine might also be delivered by inhalation. Therefore, regional concentrations might differ appreciably, especially in the pulmonary arteries and capillaries directly after central venous infusion. Finally, the sample size of our experimental groups was limited due to animal welfare regulations, which required us to use the smallest possible sample size.

In conclusion, our experiments showed that dopamine, norepinephrine, and vasopressin induce significant changes in the murine PTV. Our results suggest that they potentially modulate mucociliary clearance when applied in patients to obtain sufficient mean arterial blood pressure. Dopamine, norepinephrine, and vasopressin all initially act via substance-specific transmembrane receptors. However, they all ultimately lead to the release of intracellular Ca^2+^ from the ER, which is attenuated by additional extracellular Ca^2+^ influx. Further studies are needed to evaluate the clinical relevance of these findings and determine whether changes in mucociliary clearance could be proven in human tissues and patients during general anesthesia or critical illness when these vasopressors are used.

## Data Availability

The original contributions presented in the study are publicly available. This data can be found here: https://doi.org/10.6084/m9.figshare.26720041.v1

## Abbreviations

2-APB, 2-Aminoethoxydiphenylborane; ATP, Adenosine triphosphate; Ca^2+^, calcium ions; cAMP, cyclic adenosine monophosphate; CBF, ciliary beat frequency; DMSO, dimethyl sulfoxide; EC_50_, half maximal effective concentration; ER, endoplasmic reticulum; HEPES, 4-(2-hydroxyethyl)-1-piperazine ethanesulfonic acid; IP_3_, inositol trisphosphate; IQR, interquartile range; PKA, protein kinase A; PLC, phospholipase C; PTV, particle transport velocity; RT-PCR, reverse transcription polymerase chain reaction; SEM, standard error of the mean; SOCE, store-operated calcium entry.
